# Croup and Diphtheria

**Published:** 1877-03

**Authors:** M. P. Jacobi

**Affiliations:** 110 West 34th street


### Miscellaneous.

### Croup and Diphtheria.

Sir:—Together with many others of the medical public I have listened with great interest to your discussion at the Academy, still to be continued, on the relations between diphtheria and croup. I have been struck by the fact that, among all the opinions hitherto expressed, has not yet been presented the one held by so many European investigators: namely, that the croup that occurs in the course of diphtheria, is not a diphtheritic process, but an ordinary inflammatory process, *consequent* upon the local and constitutional conditions of the disease.*

* See Birsch Hirschfeld: Archiv der Heilkunde, 1873; Eberth; Ueber Diphtheria; Letzerich; Archiv Virch, Bd. 53; Klebs; Ueber Diphtheritis; Senator: Ueber Synanche contagiosa. Volkmann Sammlung, No. 78, 1874; Wagner; Arch. der Heilkunke, 1866. Bd. 7; Buhl Zeitschrift fur Biol., III., 1868; Rindfleisch: Path. Anat.; Waldenburg: Aerlin, klin. Wochensch, 1872.

When croup has been produced artificially in the larynx, by means of diphtheritic membranes introduced into the air-passages, the *appearance* of the croupal membrane has been identical with that of membranes formed after the introduction of chemical irritants, as ammonia.* Only in the latter case the false membranes were not contagious; in the former they proved to be so, by infecting the animals upon whom they were transported. All writers who rest the peculiarity of the diphtheritic exudations upon the presence in it of a special kind of bacteria (Micrococcus diphtheriticus, Klebs; Zygodesmus fuscus, Letzerich), admit that this parasite is never found in the larynx below the vocal cords. Senator, who denies the existence of special diphtheric bacteria, is nevertheless among those who admit a radical difference between the "fibrinous exudation in larynx and trachea," seated upon an intensely hyperæmiated mucous membrane, and the exudation in the pharynx, composed of a network of degenerated epithelium, fine granules, and micrococci, which, as the disease progresses and *inflammation is set up*, becomes a true eschar, covering an ulcerated mucous membrane, and containing detritus of its surface, together with pus cells and blood-corpuscles. "The characteristic lesion of diphtheria is an acute necrosis." "On the contrary, the croup membrane of larynx and trachea, is an exudation in the narrowest sense, consisting of fibrine and blood corpsucles,"† or, as Wagner describes it: "The so-called croup membrane consists of a thick network of delicate fibres, and of extremely numerous elements, similar to pus-corpsucles, lying in its meshes. . . . It develops by degeneration of the cylinder epithelium of the lower larynx and trachea, in the same way as the diphtheritic membrane from degeneration of the pavement epithelium of the pharynx. . . . Between the croup exudation and the surface of the mucous membrane, is a thin layer of muco-purulent fluid, and underneath, the tissues are hyperæmiated, and the seat of a moderate infiltration of pus-corpsucles and free nuclei, almost confined to the superficial layer. . . . But underneath the diphthertic exudation, the infiltration is intense, and extends to the submucosa "‡

According to Buhl, this infiltration is the most characteristic circumstance in the local process of diphtheria, and which most distinctly defines it as a "general infectious disease." The author compares the infiltration with that observed in the primitive lesions of syphilis and of tuberculosis. Croup, on the other hand, is a local inflammation—an intensification of a catarrh, *secondary* to diphtheria in most cases, even when occurring in the course of that disease. The mucous membrane is superficially infiltrated, but with pus-corpuscles, and not with elements, which, Buhl main-

---

* Oertel: Deutsch. Archiv fur klin. Med., 1871.

† Senator, loc. sit., also Buhl, loc. sit., p. 352: "After removal of the exudation there remains an ulcer with sharply jagged edges. . . . The essential part of the process is in the mucous membrane below the epithelium."

‡ Loc. cit. pp. 490–495, 496.

tains, are in diphtheria derived from conjunctive tissue cells, and tend to organize.* This doctrine corresponds to, although it be not quite identical with, the well-known distinction established by Virchow between "diphtheritic" and "croupal" inflammation.† It is much ridiculed by Sanné, author of a recent excellent systematic treatise on diphtheria.‡ "By a deplorable abuse of language," observes the French writer, "applying to pathological processes the terms which served to designate diseases, writers on the other side of the Rhine have, after the example of Virchow, called *croupal inflammation*, a phlegmasia, which, without involving the structure of the mucous membrane, deposits on its surface an exudation—a false membrane; and *diphtheritic inflammation*, an interstitial phlegmasia characterized by a sero-fibrinous exudation, which infiltrates tissues and causes their mortification. . . . Thus they have created the whimsical denominations, *croupal pneumonia*, *croupal nephritis*, on the pretext that in these cases the fibrinous exudation is effected into the pulmonary vesicles or the renal tubuli." §

Sanné considers equally *bizarre* the views of Wagner, who, "arranging pathology to suit his anatomical conceptions, maintains that a patient may have at the same time, by simple coincidence, two different diseases; diphtheria in the pharynx and supra-glottidean part of the larynx, and croup in the infra-glottidean part, and in the trachea." ‖ "The only true view is to consider diphtheria as always the same process, susceptible of assuming distinct anatomical forms according to the organ on which it is localized."

Here the author makes the very confusion between the disease and the anatomical conditions which he has so emphatically condemned. The distinction is of importance precisely in relation to the question now under discussion at the Academy, namely, the identity or non-identity of sporadic croup, and of croup occurring in the course of diptheria. The assertion of identity can mean nothing, except as regards etiology and contagion. The laryngeal lesions *are* identical as proved by the examination of both clinical and experimental cases. I have recently had an opportunity to examine the larynx and trachea in a considerable number of cases of diphtheric croup, and to compare them with a few of intense catarrhal laryngitis. In the former cases existed all the lesions of the latter, namely, fall of the epithelium, infiltration of the mucous membrane, by greater or less number of small round cells, great dilatation of blood-vessels, and frequent ecchymoses. The additional lesion in the croup was, of course, the false membrane, lying now upon parts where the epithelium was still intact, now where the mucous membrane had become denuded. The differences of

* Loc. cit., p. 367. Organizing in the roots of nerves; this same infiltration determines the diphthetitic paralysis.
† Arch. Virch., 1847, Bd. 1.
‡ Traite de la diphtherie, 1877.
§ Loc. cit., p. 34.
‖ P. 40.

opinion which concern the false membrane in the pharynx do not exist for the larynx; every one admits that it is a fibrinous exudation, whose organization into adhesions, similar to those of pleuritis and peritonitis, is only prevented by the rapid termination of the disease. "Fibrinous inflammations in general represent a more elevated degree of irritation than serous phlegmasiæ." "The blood is doubtless the principal source of the exudation, but it is not probable that this product of inflammation pre-exists in the blood. . . . We must admit that the inflamed tissues have the property of so modifying the serosity coming from the vessels, that this is changed into concrescible plasma or fibrine. This fibrinogen property would be due, according to Chalvet, to the process of denutrition which succeeds to the stage of cellular irritation, that is to say, to the materials accumulated during the *decheance* of inflamed conjunctive tissues." *

Let it be admitted that tissues, from excessive hyperæmia when the blood in the diluted vessels has been altered by diphtheritic poison would afford material possessed of more intensely irritative properties than those affected by the hyperæmia of ordinary catarrh. We should then approximatively understand the frequency of croup in diphtheria, as compared to its occurrence in non-specific pharyngitis, where the local conditions of irritation may be very much the same. But there is nothing to exclude the possibility of other causes concurring to produce the same result, namely, the communication of a fibrinogen property to the hyperæmiated epithelium or conjunctive tissues of the laryngeal mucous membrane. The question is, therefore, not Is croup diphtheria? but, admitting that diphtheria may be a cause of croup, Cannot croup be caused by something else as well?—as Oertel produced false membranes in the larynx and trachea of rabbits, by introducing on the one hand false membranes from diphtheritic patients—on the other, non-specific chemical irritants.

The distinguishing test has frequently been sought for—clinically, as in Oertel's experiments—in the capacity of these false membranes to infect other animals. I do not think that this experiment has ever been made with the false membranes derived from a case of "idiopathic" croup, although the contagiousness of this latter disease has of course been alleged as indisputable proof of its diphtheritic etiology. In the same way, the development of scarlatina in a family, immediately after a case of nephritis, awakens suspicion of the scarlatinous origin for the latter, in which a characteristic angina had been overlooked. But no one on that account would presume that nephritis could never be anything else than a symptom of scarlatina. The relations between diphtheria and croup may be aptly compared to those between

* Lancereaux, Anatomie Pathologique, p. 237, 1875. According to Buhl, the proliferating epithelium form within themselves a substance similar to fibrine, and throw it off together with the pus-corpuscles.

scarlatina and nephritis. In each case exists a specific constitutional disease with lesions characteristic or even peculiar to itself, and in each case, also, these specific lesions may be accompanied by a varying cortége of non-specific lesions, which latter may be encountered in various other groups of morbid phenomena. In each case, finally, the anatomical products of these non-specific lesions may serve to transmit the specific disease, simply because all the particles thrown off from a living body at any given time tend to reproduce themselves in their entirety, consequently with the poison with which they have become impregnated while part of that body, as soon as they can find an appropriate soil for development. Living particles from healthy bodies only tend to grow, to excite excessive local growth; hence production of pus, when they can gain admittance below the epithelium of other living bodies.* Similar particles thrown off from diseased organisms, though from parts not exhibiting the specific lesions of the disease, must similarly tend—proportionately to the degree of constitutional infection—to reproduce themselves, and with themselves the poison. Hence the inoculabifity of croupous membranes from an individual affected with diphtheria would still not prove that these were in any sense diphtheritic—still less, therefore, that false membranes in the larynx or tracnea must be proof of diphtheritic poisons.

The above remarks apply especially to the croup of children, in whom, from the smallness of the throat, the larynx lies much nearer to the seat of irritation set up by pharyngeal diphtheria, and inflammations from contiguity and inflammatory œdema are therefore facilitated. In the much rarer cases of croup complications, in the diptheria of adults, it is admitted that a true *extension* of the original diphtheritic process will much oftener be found. In the single case we have had an opportunity of examining (only microscopically), the epiglottis and entire larynx were ulcerated and covered with pultaceous exudations, entirely resembling those of the pharynx. Wagner, especially, gives a special description of laryngeal diptheria in *adults*. Besides the theoretical interest of this question, is a practical inference from the theory of croup as an inflammation induced by intense irritation of adjacent parts: we should be cautioned against irritating therapeutics, which may serve to precipitate the very lesion they propose to heroically avert.—*New York Record.* M. P. JACOBI.

110 West 34th street.

---

* Essentially the theory of Beale, which seems to us much more comprehensive than that of specific bacteria.

